# A Study to Assess the Levels of Salivary Ferritin in Iron Deficiency Anemia Subjects and Healthy Subjects

**DOI:** 10.7759/cureus.17241

**Published:** 2021-08-17

**Authors:** Lokesh Sundaram B, Vidhya Rathnavelu, Mythili Sabesan, Akila Ganesh, Soumya Anandan

**Affiliations:** 1 Medicine, Sri Ramachandra Institute of Higher Education and Research, Chennai, IND; 2 Oral Pathology and Microbiology, Sri Ramachandra Institute of Higher Education and Research, Chennai, IND; 3 Public Health Dentistry, Sri Ramachandra Institute of Higher Education and Research, Chennai, IND

**Keywords:** iron deficiency anemia, salivary ferritin, serum ferritin, correlation, potential diagnostic aid, chemiluminescence tecnique

## Abstract

Introduction: Iron deficiency anemia is one of the most widespread disorders in humans. Early diagnosis of iron deficiency anemia is challenged by assessing serum ferritin levels. However, studies across the globe have concluded that ferritin is present in quantifiable amounts in saliva. Thus, in the study, the scope of using salivary ferritin as a diagnostic biomarker in detecting iron deficiency anemia is studied.

Methods: Levels of salivary ferritin in patients with iron deficiency anemia (test group, n=15) and non-anemic subjects (control group, n=15) were assessed by an automated chemilumesent method with a total sample size of 30 volunteers.

Results: The mean level of salivary ferritin in subjects with iron deficiency anemia was 139.37±47.90 µg/dl, which was significantly higher when compared to the level in non-anemic subjects, 94.18±62.90 µg/dl, which was contradictory when compared to the levels of serum ferritin.

Conclusion: The raise in the levels of salivary ferritin in subjects with iron deficiency anemia can be attributed to the iron-dependent enzymatic function of saliva. Thus, salivary ferritin can become a biomarker that helps in the diagnosis of iron deficiency anemia; however, more research is needed for devising a more standard cutoff value for diagnosing iron deficiency anemia.

## Introduction

Iron deficiency is a common form of anemia that occurs as a result of dietary insufficiency of iron. It happens to be the most widespread disorder in humans [1]. The continued persistence of this anemia in many parts of the world is a challenge that needs to receive the highest priority in terms of attention and action. This is because the gold standard test for diagnosing anemia is assessing the levels of serum ferritin and iron, which requires drawing of venous blood [2-3]. This invasive method puts forth a number of practical and psychological complications, thus casting out a hurdle that prevents the clinicians in early diagnosis and treatment of this widespread disorder. The above-mentioned issue conveys a situation wherein the advent of newer diagnostic methods for detecting iron deficiency anemia is of prime importance.

The oral biofluid saliva, which is an ultra-filtrate of serum, is found to demonstrate an acceptable level of similarity to serum and shows changes in various systemic disease conditions that have a positive correlation with the serum [4]. Saliva shows a number of advantages including the simple and non-invasive method of collection and its easy, low-cost storage [5]. This has led to the increased research work in the field of salivaomics and use of saliva to detect various disease conditions [5].

Agarwal et al. observed that saliva contains ferritin, and that its levels in saliva were much higher than the serum levels [6]. Use of salivary ferritin can eliminate the need of drawing venous blood, thereby reducing the risk of complications, and aid in faster diagnosis of iron deficiency anemia. Hence, this study aimed to identify the scope of using salivary ferritin to diagnose iron deficiency anemia.

## Materials and methods

A comparative study was carried out to collate the levels of salivary ferritin in subjects with iron deficiency anemia and otherwise normal subjects. The study was conducted at the Department of General Medicine and Sri Ramachandra Laboratory Services, Sri Ramachandra Institute of Higher Education and Research (SRIHER), Chennai, India. This study was approved by the Institutional Ethics Committee (IEC) of SRIHER (IEC approval number CSP/19/SEP/80/321). The sample size was estimated using the Leslie Fischer formula for study populations of more than 20,000 at a 95% confidence level with a 50.0% prevalence and a degree of error set at 0.05; the sample size estimate was 323 upon normalization to an finite population; the total sample size was obtained to be 30 that was further divided into groups: Group 1 (control), subjects without iron deficiency anemia (n=15), and Group 2 (test), subjects with iron deficiency anemia (n=15).

The inclusion criteria were as follows: in Group 1, subjects without iron deficiency anemia (n=15), female volunteers 35-60 years of age, with no feature of anemia proven by serum ferritin and hemoglobin tests (serum ferritin above 12 ng/ml and hemoglobin above 12g/dl); in Group 2, subjects with iron deficiency anemia (n=15), female volunteers 35-60 years of age, with iron deficiency anemia proven by serum ferritin and hemoglobin tests (serum ferritin less than 12 ng/ml and hemoglobin less than 12 g/dl). The exclusion criteria included male volunteers, volunteers below the age of 35 and above the age of 60 and those with a history of other comorbidities and smoking habit.

After obtaining an informed consent from the subjects, 5 ml of unstimulated saliva was collected. The obtained samples were stored at -20°C. The samples were then centrifuged at 2,500 rpm for 15 minutes to remove squamous cells and cell debris. The resulting supernatant was separated into 1-ml aliquots; salivary ferritin levels were assessed by the automated chemiluminescent method. The results obtained were analysed using the dependent t-test as the statistical tool.

## Results

**Figure 1:**
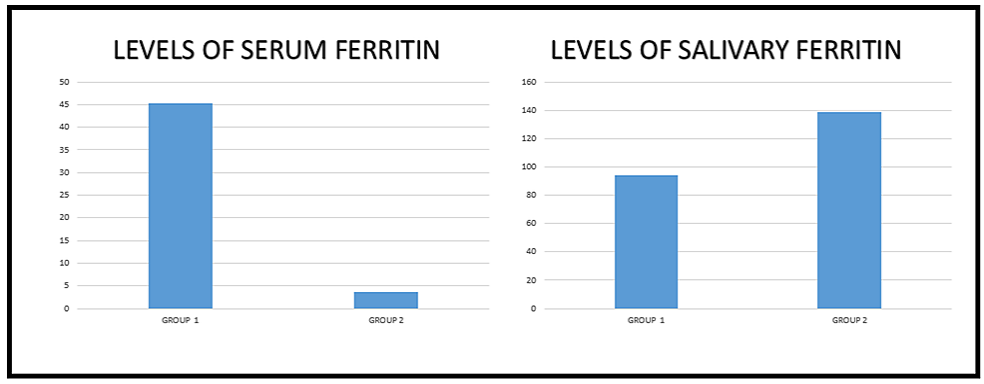
Levels of serum and salivary ferritin in control (Group 1) and test (Group 2) groups

The means age of Group 1 was 31.32±3.51 years and that of Group two was 36.26±7.28 years. Chemiluminescent analysis showed that salivary ferritin levels were higher in subjects with iron deficiency anemia (Group 2) with a mean of 139.37±47.90 µg/dl, while in the control subjects, the level measured to a mean value of 94.18±62.90 µg/dl (Group 1), which was low when compared to the subjects with iron deficiency anemia. In contrast, the serum ferritin level in Group 1 was 45.65±7.97 ng/ml, which was higher than the Group 2 mean value of 3.63±2.31 ng/ml. The t-test value for the two groups was obtained to be 1.8 (p<0.1), which was significant. The results and the demographic data of this study are given in the Table 1 and Figure 1.

**Table 1:** Ferritin levels in the two groups

Group	Age	Serum ferritin	Salivary ferritin
Group 1	31.32±3.51 years	45.65±7.97 ng/ml	94.18±62.90 µg/dl
Group 2	36.26±7.28 years	3.63±2.31 ng/ml	139.37±47.90 µg/dl

## Discussion

Iron deficiency anemia remains to prevail over 80% of the population in India and Southeast Asia [1]. It is evident from this study that there is a significant increase in the levels of salivary ferritin in patients with iron deficiency anemia. Although a significant difference in the level of salivary ferritin exists between the subjects with iron deficiency anemia and healthy subjects, the exact elucidation for such elevation in salivary ferritin is not known. However, the possible hypothesis for such an elevation is that iron is conserved in the saliva as ferritin due to the iron-dependent enzymatic function of saliva and can also be due to the endocytosis and excretion of ferritin by the salivary ducts [7-10]. Other possibilities for the increase in the salivary ferritin include the presence of large-molecular-weight iron-binding proteins and the internalization of ferritin from the intercalated ducts of parotid that can also lead to an increase in the levels of salivary ferritin [9-11].

It is also important to note that increased salivary ferritin can change the properties of saliva and also has the potential to bring about changes in the oral environment. Ferritin being acidic can reduce the salivary pH and the buffering capacity of saliva; this can in turn lead to an increased incidence of dental caries. This statement is substantiated by an epidemiological study conducted by Bansal et al. who concluded that there is an increased prevalence of dental caries in subjects with iron deficiency anemia [12]. It is also proposed that the reduction in the cytochrome oxidase level in iron deficiency anemia together with an elevation in salivary ferritin might be responsible for the occurrence of oral epithelial changes [13-14].

These findings of increased levels of salivary ferritin in subjects with iron deficiency anemia did correlate with the research work of Jagannathan et al. who conducted a similar study with pediatric patients in 2012 [2]. A similar study done by Gawaly and Alghazaly in a population of 60 Egyptian patients with iron deficiency anemia using the enzyme-linked immunosorbent assay (ELISA) test also concluded that there was an increased level of salivary ferritin in such patients [15]. The limitations of the present study were small sample size, non-inclusion of male patients, and lack of data on changes in salivary ferritin levels post-treatment. Male patients were not included in this study because iron deficiency anemia tends to be more prevalent among the female population in the chosen age group while a small sample size was chosen because of time and financial constraints.

## Conclusions

The presence of ferritin in saliva and the changes in its level in iron deficiency anemia are significant. The use of salivary ferritin as a biomarker in the diagnosis of iron deficiency can have a number of advantages including reducing the fear of blood drawn, decreased cost of the test and other technical aspects such as collection and storage of a sample. This method of detection would also be helpful during large-population epidemiological studies. However, there should be an intensive study of salivary ferritin to formulate a cutoff value to detect iron deficiency anemia.
